# Protective Coating Interfaces for Perovskite Solar Cell Materials: A First-Principles Study

**DOI:** 10.1021/acsami.1c21785

**Published:** 2022-03-04

**Authors:** Azimatu Fangnon, Marc Dvorak, Ville Havu, Milica Todorović, Jingrui Li, Patrick Rinke

**Affiliations:** † Department of Applied Physics, Aalto University, FI-00076 Aalto, Finland; ‡ Department of Mechanical and Materials Engineering, University of Turku, FI-20014 Turku, Finland; § Electronic Materials Research Laboratory, Key Laboratory of the Ministry of Education & International Center for Dielectric Research, School of Electronic Science and Engineering, Xi’an Jiaotong University, Xi’an 710049, People’s Republic of China

**Keywords:** interface, surface, level alignment, perovskite, transport layer, coating, density functional theory, Bayesian optimization

## Abstract

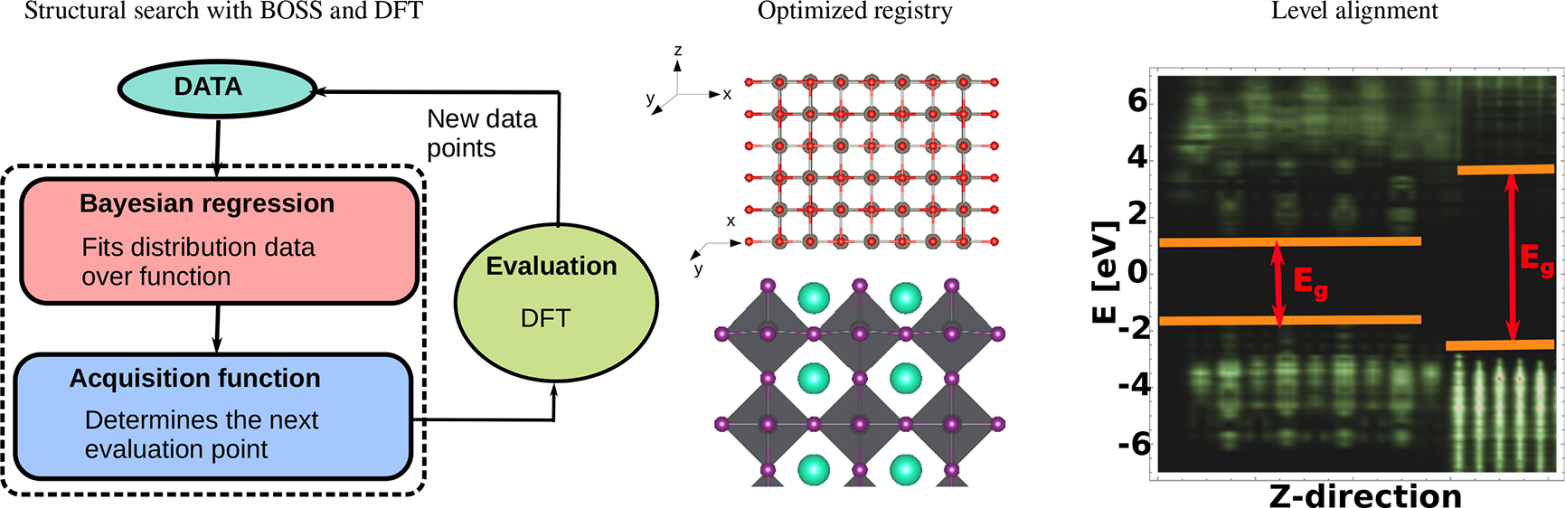

The protection of halide perovskites is important for the performance and stability of emergent perovskite-based optoelectronic technologies. In this work, we investigate the potential inorganic protective coating materials ZnO, SrZrO_3_, and ZrO_2_ for the CsPbI_3_ perovskite. The optimal interface registries are identified with Bayesian optimization. We then use semilocal density functional theory (DFT) to determine the atomic structure at the interfaces of each coating material with the clean CsI-terminated surface and three reconstructed surface models with added PbI_2_ and CsI complexes. For the final structures, we explore the level alignment at the interface with hybrid DFT calculations. Our analysis of the level alignment at the coating–substrate interfaces reveals no detrimental mid-gap states but rather substrate-dependent valence and conduction band offsets. While ZnO and SrZrO_3_ act as insulators on CsPbI_3_, ZrO_2_ might be suitable as an electron transport layer with the right interface engineering.

## 1 Introduction

Halide perovskites (HPs) have emerged as promising materials for next-generation optoelectronics, as evidenced by the steep rise in power conversion efficiency of perovskite solar cells (PSCs) from 10%^1,2^ to 25.5%^3^ within one decade. Other viable HP applications are light-emitting diodes, lasers, and photodetectors.^4−7^ HPs and HP-based devices are particularly attractive due to their ease of fabrication, low processing temperature, cost effectiveness, and availability of raw materials.^8−10^ Despite HPs’ exceptional optoelectronic properties, their large-scale production and commercialization is still impeded by several factors: the commonly used hybrid (organic–inorganic) HPs are known to suffer from rapid degradation on exposure to moisture, heat, or oxygen.^11−16^ In addition, organic charge-transport-layer materials will generally limit the device performance due to their structural and chemical disorder.^17^ For example, *Spiro*-OMeTAD, which is the most common hole-transport-layer (HTL) material in PSCs,^1,2,18−20^ suffers from instability, low hole mobility and conductivity,^21^ and undesirable effect on PSC stability.^22^ Surface passivation with more stable materials,^23−25^ especially inorganic materials, is thus important to mitigate the negative effects of ambient conditions on HP materials and devices.

These challenges could be addressed with an all-inorganic strategy.^26−28^ In this strategy, inorganic materials are chosen for all device layers: (mostly Cs-based) perovskites for the photoabsorbing or emitting layer, inorganic semiconductors as electron- and hole-transport layers, and inorganic insulators as charge-blocking materials. In this context, protective inorganic coatings have been proposed.^29−32^ It would be particularly beneficial if the coating materials could also serve as electron-transport (ETL) or hole-transport layers (HTL). So far, the studied inorganic interlayer materials in perovskite-based optoelectronic devices are mainly common binary compounds. Typical examples include TiO_2_,^33−35^ SnO_2_,^36−39^ and ZnO^34,39^ for ETLs, CuI,^40,41^ NiO,^42−45^ graphene oxide,^46^ and CuSCN^45^ for HTLs, and alkali-metal halides for charge-blocking layers.^47−50^

**Figure 1:**
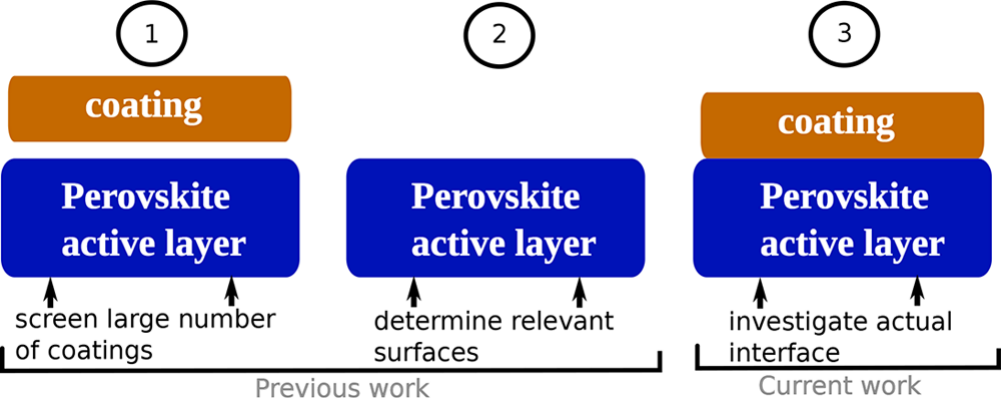
Conceptual workflow of identifying and characterizing suitable perovskite coating materials.

In our previous work, we applied a data-driven approach to discover inorganic materials suitable for perovskite-based devices with the aim of further enhancing the device performance and stability.^32^ We developed a three-stage scheme as shown in Figure 1. At stage 1, we screened a materials database for inorganic coating materials that meet a series of requirements such as band gap, stability, transport properties, and crystal structure.^32^ Stage 2 identifies the stable surface structures of CsPbI_3_ and CH_3_NH_3_PbI_3_ under different growth conditions, for which we carried out a surface-phase-diagram analysis based on density-functional-theory (DFT) and *ab initio* thermodynamics.^51,52^

This work presents stage 3, which computationally estimates whether the materials that pass the screening at stage 1^32^ are indeed good coating candidates for HPs. To this end, we investigate the interfaces between the candidate coatings and a series of HP surface models produced at stage 2^51^ using DFT. The surface registry match between the perovskite and the coating was determined by Bayesian optimization (BO) active learning, based on the minima in the binding energy landscape. The minima of the energy landscape corresponds to the binding energy (*E*_b_) of the most stable registry. We chose α-CsPbI_3_ as the prototype HP model system for this first case study, while similar systems such as γ-CsPbI_3_ and CH_3_NH_3_PbI_3_ will be the subject of future work.

The remainder of this paper is organized as follows. We present a brief description of our computational approach in Section 2. In Section 3, we present the results from the BOSS/DFT calculations, analyze the features of the optimized interface structures, and establish the level alignments at the coating–perovskite interface for all coatings and substrates. Section 4 presents a brief discussion of our results. We conclude with a summary of our findings in Section 5. Computational details are presented in Section 6.

## 2 Computational Approach

**Figure 2:**
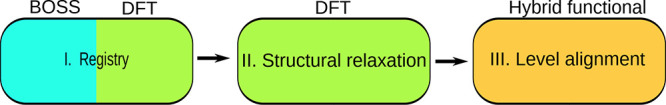
Protocol for computational characterization of coating–perovskite interfaces with BOSS and DFT.

We designed a three-step protocol for our interface study, as sketched in Figure 2. Step I employs the recently developed Bayesian Optimisation Structure Search (BOSS) package^53^ to determine the registry between the coating and the HP substrate. With this registry as the starting point, the atomic positions of the coating–perovskite interface are relaxed with DFT in step II. In step III, interfacial electronic structure calculations are carried out for the relaxed interfaces using hybrid functional DFT.

We propose such a two-step (steps I and II) structure-search strategy on the basis of the following considerations. The most stable interface geometry corresponds to the global minimum of the total-energy landscape of the coating–perovskite combination, which is a multidimensional function of a series of parameters that define the relative geometry between these two components. This total-energy landscape is very complex due to the polyatomic nature of both the coating and HP surfaces. Therefore, it is not easy to identify the most stable structure with a simple DFT structure relaxation, as different initial structures might fall to different local minima and miss the global minimum.

BOSS has already shown its power in problems such as a conformer search for organic molecules,^53,54^ organic molecule adsorption at semiconductor surfaces,^53^ and film growth of organic adsorbates on metallic surfaces.^55−57^ In this work, we employ BOSS to tackle the interface problem between two inorganic materials (coating and HP) in step I, which we believe can efficiently narrow down the search space for further geometry relaxation in step II. In our BOSS search, state-of-the-art single-point DFT calculations are performed for different structures, and BOSS correlates the structures with an energy landscape through active learning with a BO algorithm. A surrogate model is fitted to the DFT data points employing Gaussian process regression (GPR), which is refined by acquiring further data with a smart sampling strategy. In such a way, a relatively modest number of DFT data points suffice to converge the multidimensional binding energy landscape.

For a semi-infinite slab with a thin coating layer, the band gap deep in the bulk is the same for any surface reconstruction or defect. For this reason and to provide a consistent ranking of level alignments among coatings, we use the bulk as a “model substrate” for all the interfaces. For each interface, we extracted the valence (VB) and conduction band (CB) offsets from the spatially resolved local density of states (LDOS) of Figure S5 in the Supporting Information. The offsets are then added (or subtracted) from the bulk conduction band minimum (CBM) (or valence band maximum, VBM) to create the alignments here. Our bulk band structure and LDOS plots are based on a hybrid functional. We also included spin–orbit coupling (SOC) in the bulk band structure calculation. On the basis of our previous works, we expect the inclusion of SOC to shift the CBM down (into the gap), which will lead to a reduction of the band gap energy. To account for the changes resulting from SOC, we will shift the VBM and CBM of the bulk LDOS for our final coating–perovskite interface level alignments. Details of these calculations are outlined in Section 6.

## 3 Results

In this work, we find the stable registry and interface structure, analyze the electronic properties, and establish the level alignment at the coating–perovskite interfaces. We considered SrZrO_3_ (cubic, *Pm*3̅*m*), ZnO (cubic, *Fm*3̅*m*), and ZrO_2_ (tetragonal *P*4_2_/*nmc*) as coatings on the basis of results from our previous study.^32^ For the substrate, we investigated both the ideal clean CsI-terminated (CsI-T) and reconstructed surface models with adatoms (of CsPbI_3_) to simulate different synthesis conditions. The selected surface reconstructions are taken from ref (51), in which they were determined to be the most relevant reconstructions. We used the stable reconstructed surface models clean CsI-T surface, i_PbI_2__, i_2PbI_2__, and i_4CsI_ of *Pm*3̅*m* CsPbI_3_. *i*_X_ denotes adatom structures with X = PbI_2_, 2PbI_2_, 4CsI. Details of the computations are outlined in Section 6. This section will present and discuss the binding energy landscapes and optimized interface structures for ZnO on the four substrates as a prototype. We use ZnO as a prototype because, of all the coatings in this work, it is the most studied transport layer in PSCs. Similar results for the other coatings are presented in the Supporting Information. The level alignments at the coating–perovskite interface for all of the structures in this work will also be presented and discussed.

### 3.1 Binding Energy Landscapes from BOSS

**Figure 3:**
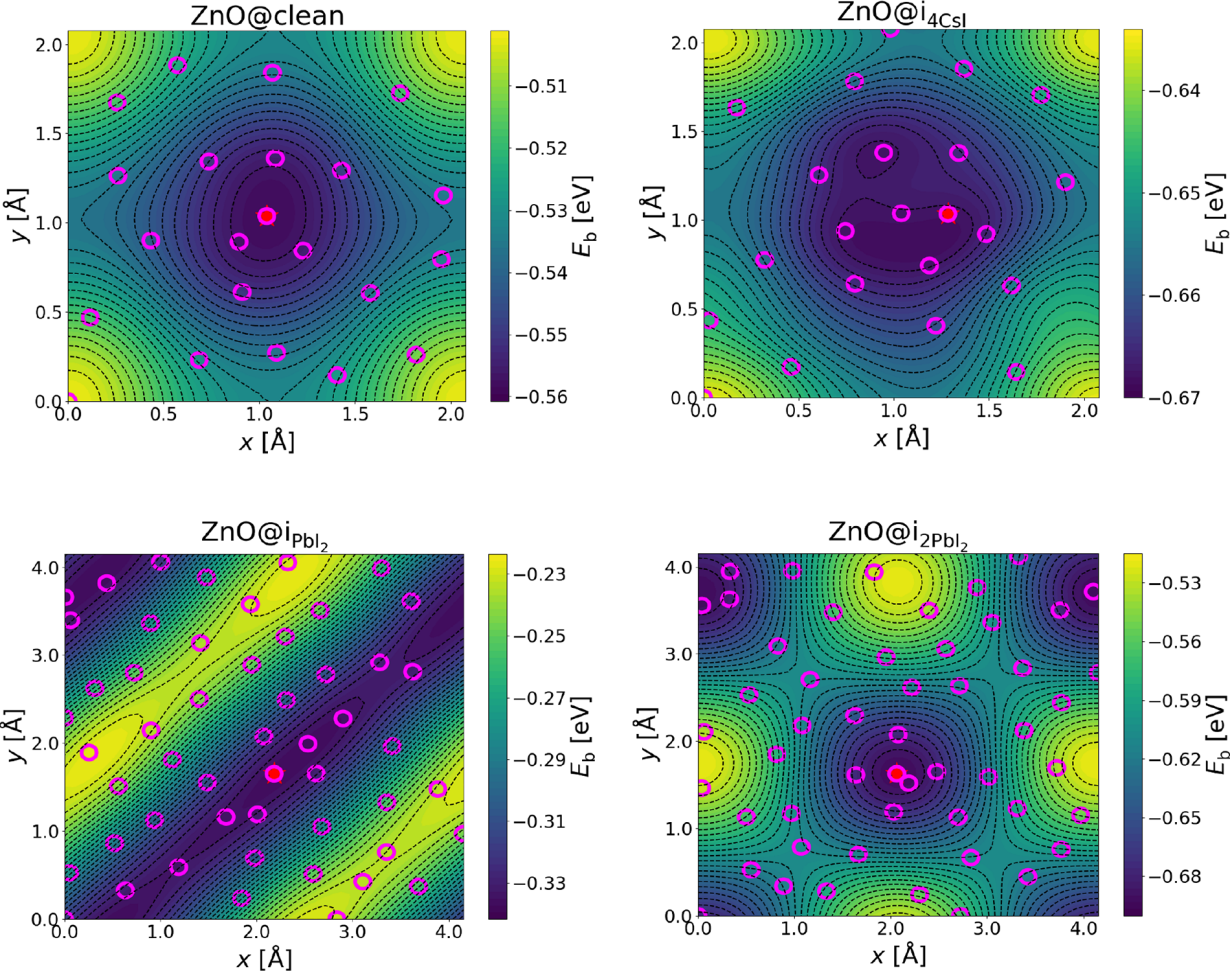
Binding energy landscapes of perovskite–coating with BOSS. The pink circles and red stars depict the acquisition points and minimum potential energies, respectively, as the coating is translated by *x*, *y* on the substrate.

Figure 3 shows the two-dimensional (2D) binding energy landscapes for ZnO@clean, ZnO@i_PbI_2__, ZnO@i_2PbI_2__, and ZnO@i_4CsI_. The 2D binding energy landscapes for SrZrO_3_ and ZrO_2_ on the four substrates are shown in Figure S1 in the Supporting Information. The pink circles depict the acquisition points and the red stars the minimum potential energies, which corresponds to the minimum binding energy (*E*_b_) of the surrogate models. The yellow shades in the color map depict regions of high binding energy followed by green shades, with the dark blue shades showing the lowest energies. Here, *x* and *y* are the translations of the coating in the *x* and *y* directions from some initial origin. Due to the periodicity of the system in *x* – *y*, these displacements describe the entire search domain.

The binding energy landscapes for ZnO@clean and ZnO@i_4CsI_ (top panels in Figure 3) exhibit an *x* and *y* range of 0.00–2.08 Å, representing the smallest unit cell of the coating@perovskite interface. For a 3 × 3 × 3 coating on a 2 × 2 substrate, the primitive unit cell of the ensemble is one-sixth of the supercell, which corresponds to a search space of 0.00–2.08 Å. In the case of ZnO@i_PbI_2__ and ZnO@i_2PbI_2__, the interface commensurability changes due to the added PbI_2_ complexes. For these, we used a search space of one-third of the supercell corresponding to 0.00–4.15 Å. The registries with the lowest binding energies are given in Table S2 in the Supporting Information.

Due to the similar search spaces for ZnO@clean and ZnO@i_4CsI_, the binding energy landscapes are almost the same and their minimum energies (red stars) are both located at the center of the landscape. In ZnO@i_PbI_2__, the landscape looks different due to the broken periodicity emanating from the added PbI_2_ unit. Similarly, the pattern in ZnO@i_2PbI_2__ is different. Here, a diagonal periodicity is seen due to the repeated PbI_2_ unit on the surface of the substrate.

### 3.2 Optimized Interface Structures

**Figure 4:**
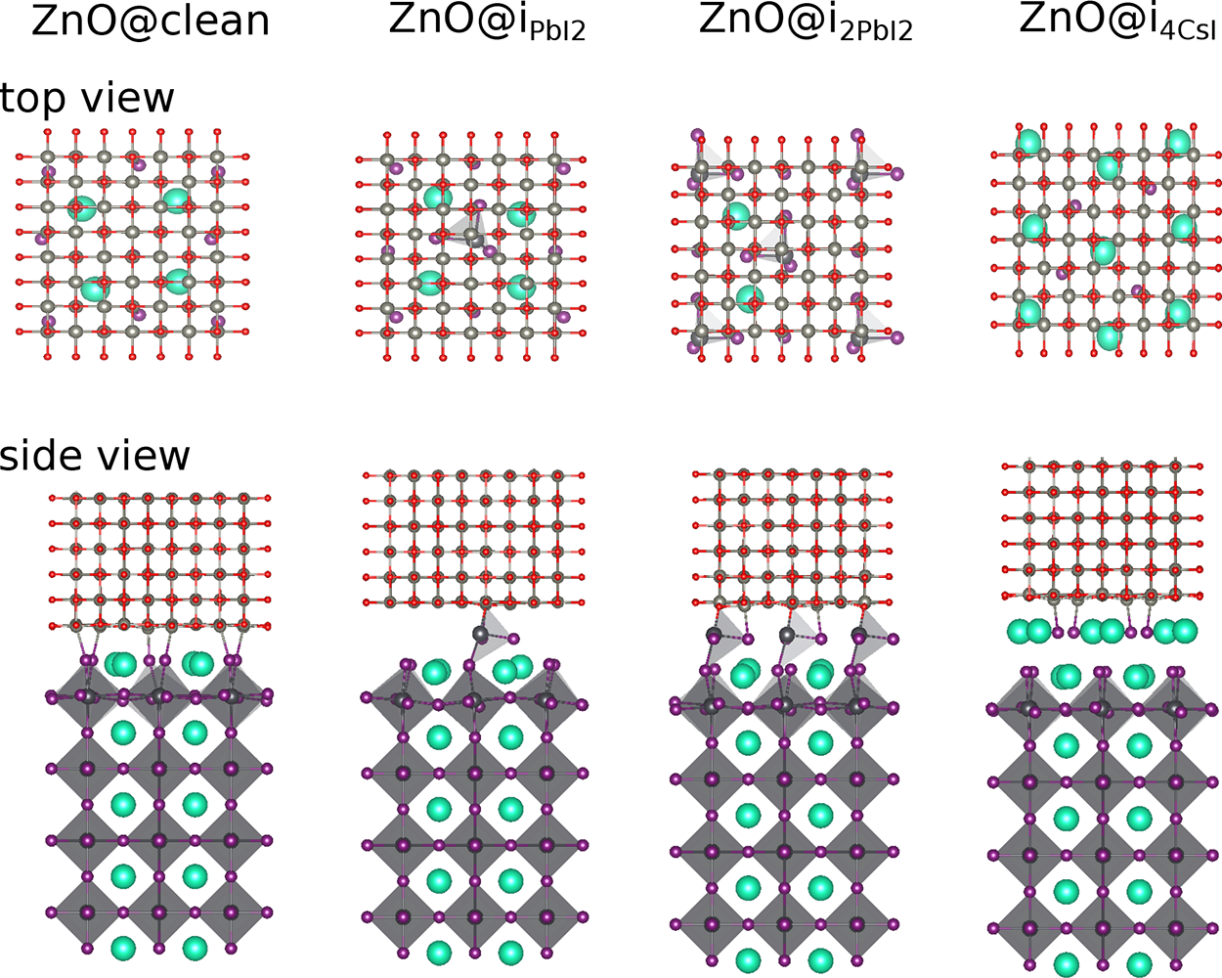
Optimized structure of ZnO on the clean surface and its reconstructed models: from left to right are ZnO@clean, ZnO@i_PbI_2__, ZnO@i_2PbI_2__, and ZnO@i_4CsI_. Cs, Pb, I, Zn, and O are displayed in green, black, purple, light gray, and red, respectively. The PbI_6_ octahedra are shown in dark gray.

Figure 4 depicts the optimized interface structures obtained from DFT relaxations of the optimal registry positions for ZnO@clean, ZnO@i_PbI_2__, ZnO@i_2PbI_2__, and ZnO@i_4CsI_. Similar results for SrZrO_3_ and ZrO_2_ are shown in Figure S2 in the Supporting Information. Detailed structural and computational information are outlined in Section 6. We observe a rearrangement of atoms in the substrates to accommodate the lattice strain between the coatings and substrates. Specifically, the Cs–I bond lengths in the topmost substrate layers change slightly to accommodate the bonding between the bottom layers of the coatings and the CsI layers. This is more pronounced in ZnO@i_4CsI_, where the topmost CsI layer is pulled into the coatings. Similarly, the Pb–I bonds in the topmost polyhedra of ZnO@i_PbI_2__ and ZnO@i_2PbI_2__ tilt slightly at the interface. We also observe similar features for ZrO_2_- and SrZrO_3_-based interfaces (Figure S2). Despite these changes in bond lengths, our coatings show strong bonding with the substrates with no visible structural distortions.

Table 1 gives the optimized binding energies and lattice strains for all three coatings on our four most relevant reconstructed α-CsPbI_3_ surface models. Here, *E*_b_ = *E*_ensemble_ – (*E*_coating_ + *E*_substrate_). The absolute lattice strain increases from SrZrO_3_ to ZrO_2_, with ZnO having the largest value. Similarly, the binding is reduced with an absolute increase in lattice strains (i.e., the absolute value of the binding energy decreases). Specifically, substrates with SrZrO_3_ coatings show the strongest binding followed by those with ZrO_2_ and ZnO coatings (Table 1). This observation can be simply explained by the fact that structures with larger mismatch have a greater energetic cost due to strain, hence leading to a decrease in binding strength. Figure S3 in the Supporting Information shows a plot of binding energies as a function of lattice strain. Structurally, we also observe a minimum rearrangement in atomic positions for ZnO and ZrO_2_ on all substrates. SrZrO_3_, on the other hand, exhibits significant rearrangement, causing shifts in atomic positions (Figure S2). These observations could also contribute to the varying binding energies, as seen in Table 1.

**Table 1:** Binding Energies (in eV) and Lattice Strains (in Percent) of the Three Coatings on the Four Most Relevant α-CsPbI_3_ Reconstructed Surface Models

		binding energy			
	strain	clean	i_PbI_2__	i_2PbI_2__	i_4CsI_
SrZrO_3_	1.0	–5.53	–5.38	–7.03	–8.16
ZrO_2_	–2.2	–2.14	–3.14	–4.85	–4.23
ZnO	4.5	–1.15	–0.73	–1.32	–2.11

### 3.3 Level Alignment of Coating–Perovskite Interfaces

**Figure 5:**
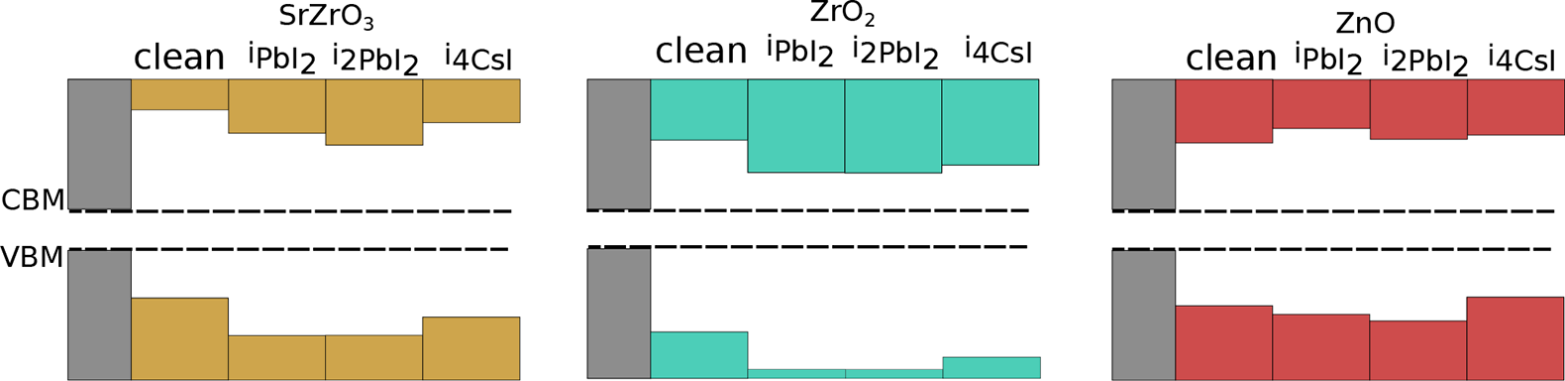
Band alignment at the coating–perovskite interface. The gray, red, yellow, and green shaded regions are representative of the bulk substrate, ZnO, SrZrO_3_, and ZrO_2_, respectively. The dashed lines depict the valence and conduction band edges. Here, the VBM and CBM are set to the bulk values of CsPbI_3_ calculated with a hybrid functional including SOC.

Figure 5 summarizes the level alignments for all interfaces investigated in this work. Our band structures and LDOS plots (Figure S4–S6 in the Supporting Information) exhibit no mid-gap states. Table 2 summarizes the band offsets for all interfaces. Upon the inclusion of SOC in the bulk band structure calculation, the CBM is pulled into the gap by ∼0.8 eV while the VBM shifts up by ∼0.1 eV, reducing the bulk band gap energy to 1.34 eV. To account for the effect of SOC in our coating–perovskite interface level alignments, we shifted the VBM and CBM of the bulk LDOS by 0.1 and −0.8 eV, respectively. The band structures and LDOS of all interfaces in this work are shown in Figures S4 and S5, respectively.

**Table 2:** Valence (VB) and Conduction Band (CB) Offsets (in eV) at the Coating–Perovskite Interfaces

	band offset							
	clean		i_PbI2_		i_2PbI2_		i_4CsI_	
	VB	CB	VB	CB	VB	CB	VB	CB
SrZrO_3_	1.46	3.05	2.62	2.33	2.61	1.96	2.05	2.65
ZrO_2_	2.56	2.12	3.71	1.12	3.72	1.11	3.33	1.35
ZnO	1.69	2.03	1.96	2.48	2.16	2.15	1.43	2.28

In all cases, we observe a type I level alignment (straddling gap). Of all three coatings, ZrO_2_-based interfaces show the largest VB offsets with ZrO_2_@i_PbI_2__ exhibiting the largest offset. Concomitantly, the CB offsets are the smallest. Conversely, ZnO-based interfaces (except for ZnO@i_PbI_2__) show larger CB offsets. Interestingly, we observe a mixed trend in SrZrO_3_-based interfaces. Specifically, SrZrO_3_@clean and SrZrO_3_@i_4CsI_ show larger CB to VB offsets while the opposite is seen in SrZrO_3_@i_PbI_2__ and SrZrO_3_@i_2PbI_2__.

## 4 Discussion

Despite a noticeable atomic displacement in the topmost layers of the substrates (Figure 4), Figure S4 and S5 in the Supporting Information show no electronic states in the band gap. The absence of such gap states is beneficial for devices, since they could act as nonradiative recombination sites. We cannot, however, exclude the presence of structural defects in real devices that introduce such gap states.

The different band alignments in Figure 5 show that the interface can be engineered to enhance charge collection or blocking. Studies have shown that ETLs (and HTLs) with a wide band gap and small CB (VB) and large VB (CB) offsets to the substrates have the potential to efficiently fulfill exciton confinement and hole-blocking (electron-blocking) functions in PSCs.^2,33^ ZnO is known to be a wide-band-gap (>3 eV) n-type semiconductor that has been widely explored in optoelectronics.^34,58−60,60−62^ SrZrO_3_ is also an intrinsic perovkite semiconducting material, which means that it has the tendency to transport both electrons and holes. Conversely, ZrO_2_ is a well-known insulating material that has been used as a protective coating in optoelectronics.^63,64^

Our results indicate that ZrO_2_ might not only act as insulating layer on CsPbI_3_ but could also be engineered to be an ETL. The CB offsets are the lowest we observe for all interfaces in this work, and additional iodine in the form of PbI_2_ or CsI reduces the CB offsets considerably in comparison to the clean interface. This suggests that further interface modifications might lower the CB offsets sufficiently for ZrO_2_ to become an ETL.

## 5 Conclusion

In summary, we have successfully studied the interactions of the coating materials ZnO, SrZrO_3_ and ZrO_2_ on four reconstructed CsPbI_3_ surface models (clean, i_PbI_2__, i_2PbI_2__, and i_4CsI_) by combining a machine-learning-based structure search method and DFT. Our optimized structures show strong bonding between the coatings and the substrates at the interfaces. Despite the changes in the atomic positions at the topmost layers of our substrates, our spatially resolved local density of states analysis exhibits no mid-gap states, which is good for transport properties across the interfaces. We further observed that both the VB and CB offsets for all coatings are large. ZrO_2_ exhibits the smallest CB offset and could potentially, with the right interface engineering, serve as an ETL. Our current and previous studies serve as a starting point for future work on surface adsorbates, defects, and interface engineering of PSCs.

## 6 Computational Details

### 6.1 Interface Registry Search with BOSS-DFT

Data acquisitions that serve as inputs to BOSS are the binding energies (*E*_b_) of single-point calculations at the relative perovskite–coating shift (*x*, *y*) suggested by the BOSS acquisition function. The atomic structures of the coating and perovskite are kept fixed. With each additional sampled configuration, Gaussian process models for the energetics are fitted with an uninformative prior on the mean and the model hyperparameters are optimized following the standard procedure of maximizing the log marginal likelihood. We employed the exploration-biased Lower Confidence Bound (eLCB) acquisition function, which balances exploitation against exploration.^76^ The procedure is iterated until the BOSS surrogate model converges (20 iterations of the coatings on clean and i_4CsI_, 50 for coatings on surfaces with added PbI_2_ units). We monitor convergence by tracking the minima of *E*_b_ within [−3:0] eV. We then extract the global minimum from the BOSS surrogate model and use the structural model at the corresponding (*x*, *y*) coordinates as an input for DFT geometry optimization.

### 6.2 Interface Structural Information

We used BOSS and DFT to search for the optimal configurations of our perovskite–coating interfaces. For all of our coating materials, we used a 3 × 3 × 3 supercell, which is close to being commensurate with a 2 × 2 CsPbI_3_ substrate. To facilitate DFT interface calculations with periodic boundary conditions, we adjusted the lattice constants of the coatings (ZnO, *a* = *b* = *c* = 13.02 Å; SrZrO_3_, *a* = *b* = *c* = 12.59 Å; ZrO_2_, *a* = *b* = 12.19 Å and *c* = 21.57 Å) slightly such that the three coating unit cells fit exactly onto two CsPbI_3_ unit cells (i.e., are 12.46 Å long). In this work, we chose the cubic structure of ZnO due to its commensurabililty with the substrate even though the wurtzite structure is the most common polymorph used in optoelectronics. However, studies have shown^65−69^ that, in real device engineering, the reduction in size of inorganic materials to the nanoscale induces different structural ordering relative to the most stable bulk polymorph. The perovskite surface models used as substrates in this work are the most stable reconstructed surfaces (clean, i_PbI_2__, i_2PbI_2__ and i_4CsI_) of α-CsPbI_2_ from our previous work.^51^

### 6.3 Boundary Conditions for Structural Search

**Figure 6:**
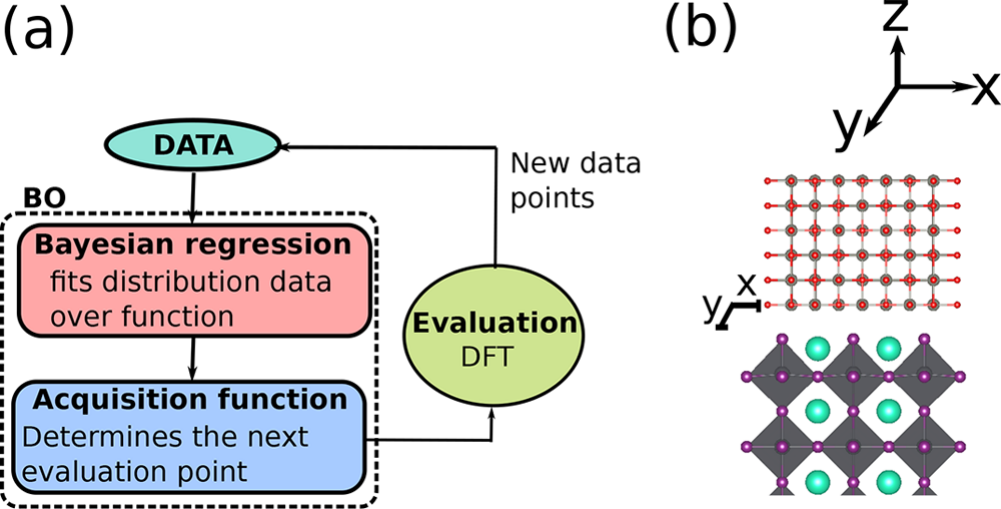
Workflow of the BOSS structural search and an example of its performance: (a) principles governing the buildup of a surrogate model by BOSS; (b) registry of the structure under investigation from which the binding energy is determined by varying *x* and *y*.

Figure 6a shows the three-step approach for the structural search with BOSS and DFT. Figure 6b depicts the registry for ZnO on the CsI-T surface model. *x* and *y* are the translations of the coating in the *x*–*y* plane away from an arbitrary initial origin ([0,0]) which corresponds to a search domain *x*,*y* ∈[0.0–2.08] Å for coatings on the clean and i_4CsI_ substrates. For coatings on substrates with added PbI_2_ units, the search domain corresponds to *x*,*y* ∈[0.0–4.15] Å. By symmetry and with our choice of origin, the translations of the coating in *x* and *y* are equivalent.

In all of our structural models, we kept the distance between the coating and the perovskite along the *z* axis constant at ∼4 Å on the basis of preliminary investigations with a three-dimensional search space. In our case, the *z* direction is not as important, since our tests show that it does not affect the shape or features of the 2D energy landscape and the final relaxation corrects the bonding of the atoms at the interface.

### 6.4 DFT and Band Alignment Computations

To overcome a numerical error from BOSS, which does not enforce symmetry, we averaged the optimal *x* and *y* translations at the local minima obtained from the BOSS/DFT run before relaxation. By fixing the bulk units (all layers below the topmost CsI and PbI_2_ units) of the substrates, we then relaxed these structures, including the out-of-plane distance *z*, and calculated the spatially resolved local density of states (LDOS) using the hybrid Heyd–Scuseria–Ernzerhof (HSE06, simplified as HSE in this paper)^70^ functional. From the HSE calculations, we deduced the band alignments at the coating–perovskite interfaces.

We used the PBEsol^71^ functional with tier-1 basis sets for the single-point BOSS/DFT calculations and tier-2 basis sets for the structural relaxation (as implemented in the FHI-AIMS code^70,72^). In all cases, we used a Γ-centered 4 × 4 × 1 *k*-point grid. We also included dipole corrections^73^ and a vacuum size of ∼40 Å to avoid dipole interactions between neighboring slabs.

In our HSE calculations, we used the standard range-separation parameter ω = 0.11 bohr^–1^ but adjusted the amount of exact exchange (α) to 0.55. We also included spin–orbit coupling in our bulk band structure calculations to establish the effect of band splitting on the electronic properties. The α value was obtained by fitting our HSE+SOC band gap to the GW band gap (*E*_g_= 1.48 eV) of the cubic (α) CsPbI_3_ structure reported in ref (74) (see the Supporting Information for details).

## Supporting Information Available

Energy landscapes of SrZrO_3_ and ZrO_2_, binding energies of the interfaces before relaxation, optimized interface structures of SrZrO_3_ and ZrO_2_, a plot of the optimized binding energies of all interfaces as a function of lattice strain, and the band structures and spatially resolved density of states (LDOS) of all interfaces (PDF)
