# Supplementary material for: Protective Coating Interfaces for Perovskite Solar Cell Materials: A First-Principles Study
Source: ACS Appl Mater Interfaces. 2022 Mar 4;14(10):12758–65. doi: 10.1021/acsami.1c21785 (PMC8931722; doi:10.1021/acsami.1c21785)
Supplement: Supplementary file 1 — am1c21785_si_001.pdf [file am1c21785_si_001.pdf]

Supplementary Information

Protective Coating Interfaces for Perovskite Solar Cell Materials: A First Principles Study

Azimatu Fangnon,*,† Marc Dvorak,† Ville Havu,† Milica Todorović,‡ Jingrui Li,¶ and Patrick Rinke†

†*Department of Applied Physics, Aalto University, FI-00076 AALTO, Finland*

‡*Department of Mechanical and Materials Engineering, University of Turku, FI-20014 Turku, Finland*

¶*Electronic Materials Research Laboratory, Key Laboratory of the Ministry of Education & International Center for Dielectric Research, School of Electronic Science and Engineering, Xi'an Jiaotong University, Xi'an 710049, China*

E-mail: azimatu.fangnon@aalto.fi

S-1 Binding energy landscapes from BOSS-DFT structural search

Figure S1 shows the energy landscapes for $SrZrO_3$ and ZrO_2 on the α-$CsPbI_3$ most stable reconstructed surface models (clean, i_{PbI_2}, i_{2PbI_2} and i_{4CsI}). x, y and E_b depict the varied x-, y-coordinates and the potential energy at a given x, y pair, respectively. The pink circles and red star are representative of the acquisition points and the optimal potential energy at x and y respectively.

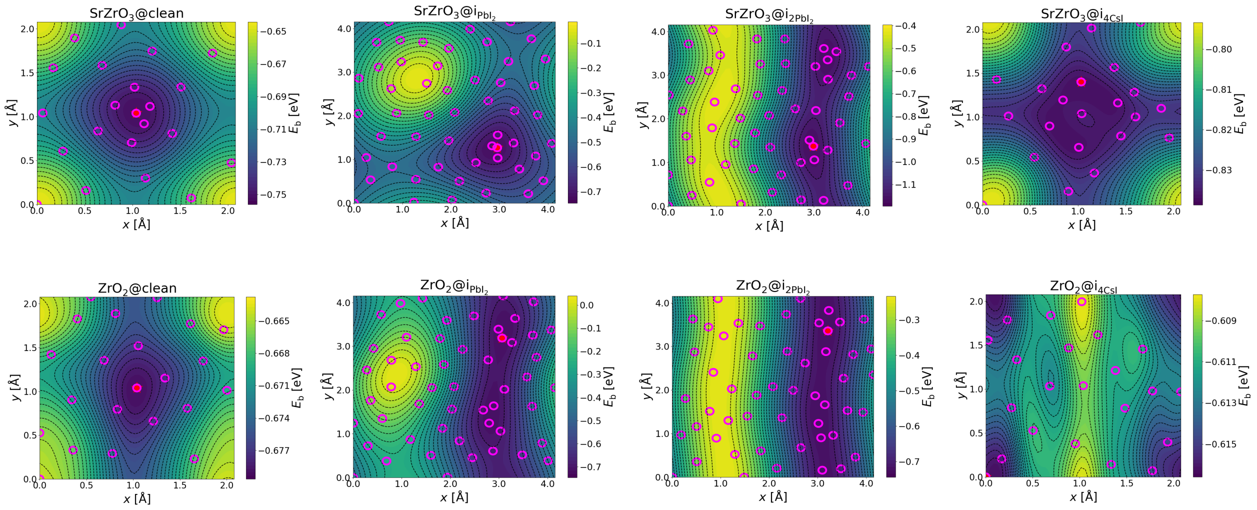

Figure S1: Energy landscapes of perovskite-coating interfaces with BOSS and DFT. The pink circles and red star depict the acquisition points and minimum potential energies at x and y respectively. The top panel consist of $SrZrO_3$ on CsI-T, i_{PbI_2}, i_{2PbI_2} and i_{4CsI}. The bottom panel comprises of ZrO_2 on CsI-T, i_{PbI_2}, i_{2PbI_2} and i_{4CsI}.

Table S1 lists the minima of the surrogate models from the BOSS/DFT search (i.e. positions of the red stars of Fig. S1).

Table S1: Minimum binding energies (in eV) and their corresponding locations (acquisition points) (x, y) of the surrogate model from the BOSS/DFT structural search.

	$SrZrO_3$			ZrO_2			ZnO		
	Location [Å]		E_b	Location [Å]		E_b	Location [Å]		E_b
	x	y	[eV]	x	y	[eV]	x	y	[eV]
clean	1.04	1.04	−0.75	1.04	1.04	−0.68	1.04	1.04	−0.56
i_{PbI_2}	2.96	1.27	−0.72	3.09	3.19	−0.72	2.18	1.65	−0.34
i_{2PbI_2}	2.99	1.37	−1.17	3.21	3.36	−0.72	2.06	1.63	−0.69
i_{4CsI}	3.08	2.78	−0.84	0.00	0.00	−0.62	0.76	3.17	−0.67

S-2 Optimized interface structures

Figure S2 depicts the optimized atomic structures of $SrZrO_3$ (top panel) and ZrO_2 (bottom panel) on CsI-T, i_{PbI_2}, i_{2PbI_2} and i_{4CsI}, respectively.

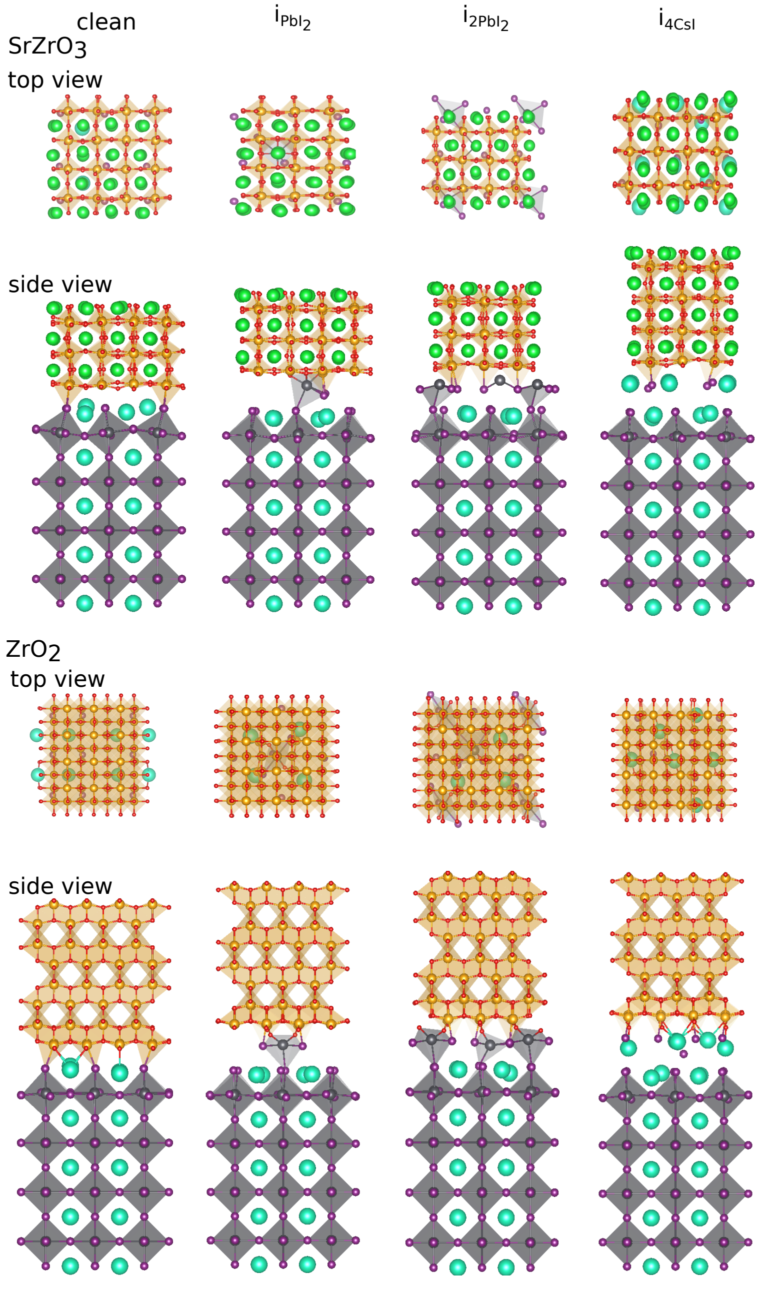

Figure S2: Optimized coating/perovskite interfaces for $SrZrO_3$ (top panel) and ZrO_2 (bottom panel on CsI-T, i_{PbI_2}, i_{2PbI_2} and i_{4CsI}.

S-3 Binding energies of optimized structures vs lattice strain

Figure S3 shows the binding energies of the optimized structures as a function of lattice strain. Coatings on the clean surface, i_{PbI_2}, i_{2PbI_2} and i_{4CsI} are depicted by black, red, blue and pink colours, respectively. The x-axis shows the absolute value of the lattice mismatch between the substrate and each coating.

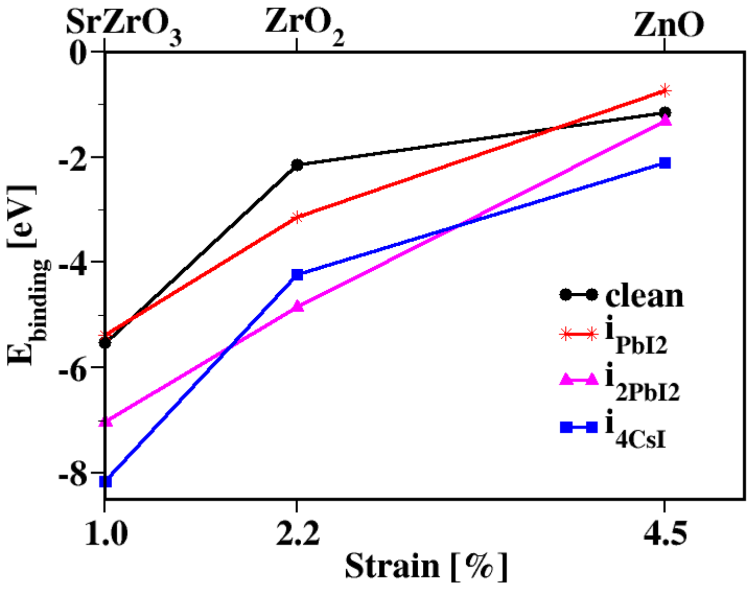

Figure S3: Binding energies of optimized structures as a function of lattice strain. Coatings on clean surface, i_{PbI_2}, i_{2PbI_2} and i_{4CsI} are depicted by black, red, blue and pink colours respectively. The x-axis shows the absolute value of the lattice strain of each coating and the y-axis the binding energy for that substrate-coating pair.

S-4 Estimation of the hybrid exchange correlation coefficient

We estimated the hybrid exchange correlation coefficient (α value) by fitting our HSE+SOC band gap to the *GW* band gap of Ref. 74. This we achieved by using the bulk cubic unit cell provided in Ref. 74. The lattice constants of the Reference bulk unit cell are $a = b = c = 6.30$ Å. We varied the α value from $0.1 - 0.6$ as shown in Fig. S4. The dash red line depicts the estimated α value of 0.55 which corresponds to a band gap energy of ~ 1.48 eV.

In our work, we reported an HSE+SOC bulk band gap energy of ~ 1.34 eV, which differs from the Reference value by ~ 0.14 eV. This difference can be attributed to the difference in the lattice constants of the Reference structure and our PBEsol optimized bulk structure ($a = b = c = 6.22$ Å).

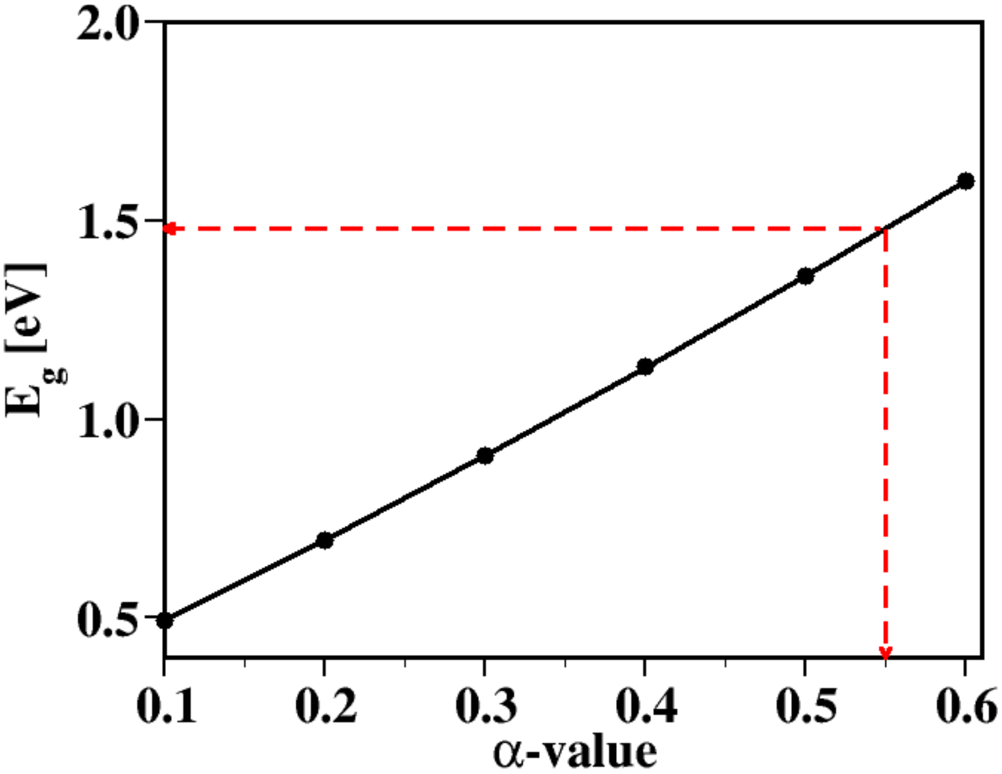

Figure S4: Estimated hybrid exchange correlation coefficient (α value for HSE band structure calculation. The dash red line depicts the estimated α value (on the horizontal axis) and its corresponding band gap energy (on the vertical axis).

S-5 Band structures of bulk and all interfaces

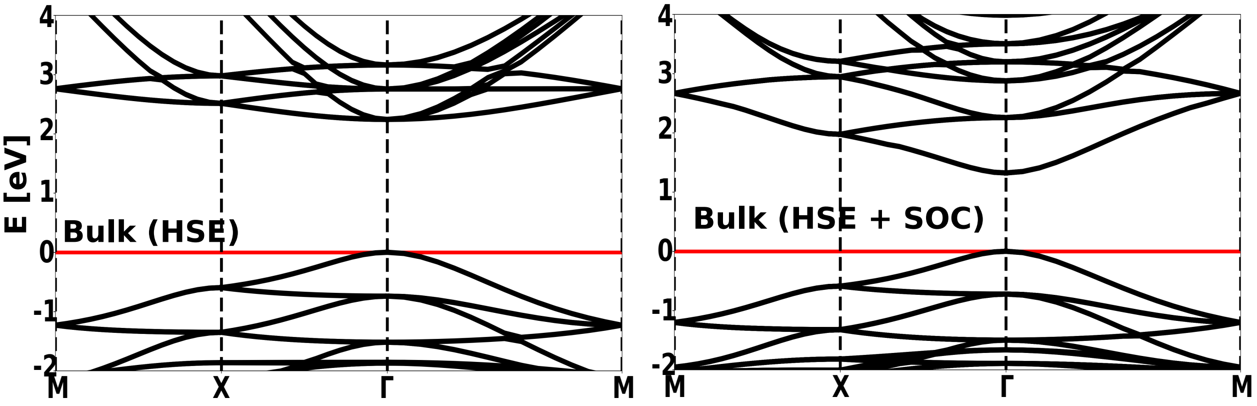

Figure S5: Bulk band structure with and without spin-orbit coupling (SOC). The red horizontal line is representative of the valence band maximum.

Figure S5 shows the bulk band structure calculated with (right panel) and without (left panel) spin-orbit coupling (SOC). The red horizontal line depicts the valence band maximum (VBM). Upon inclusion of SOC, the conduction band minimum (CBM) is shifted down by ~ 0.8 eV while the VBM shifts up by ~ 0.1 eV, reducing the bulk band gap energy to 1.34 eV.

Figure S6 shows the band structures of ZnO, $SrZrO_3$ and ZrO_2 on the α-$CsPbI_3$ reconstructed surface (CsI-T, i_{PbI_2}, $i_{2\mathrm{PbI}_2}$ and $i_{4\mathrm{CsI}}$) models. The red horizontal depicts the valence band maximum (VBM). The band structures of the coating-perovskite interface are overlayed on to the clean surface model for ease of comparison.

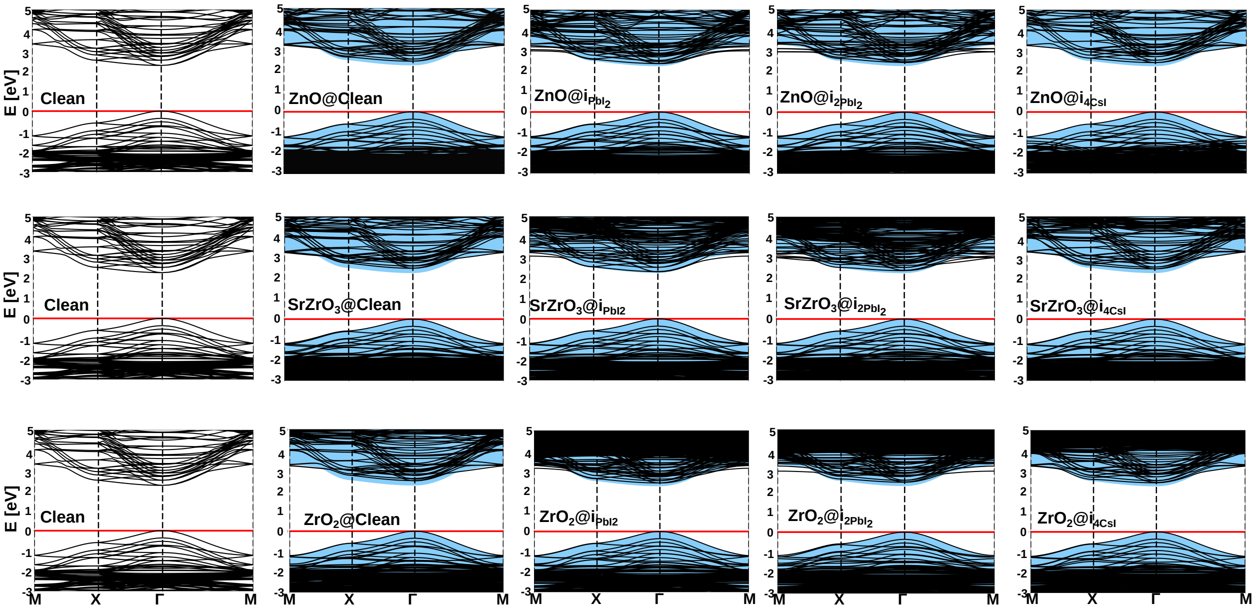

Figure S6: Band structures for ZnO, $SrZrO_3$ and ZrO_2 on the α-$CsPbI_3$ reconstructed surface (CsI-T, i_{PbI_2}, i_{2PbI_2} and i_{4CsI}) models. The red horizontal depicts the valence band maximum (VBM) The band structures of the coating-perovskite interface are overlayed on to the clean surface model for easy of comparison.

S-6 Spatially resolved local density of states and band alignment

Figure S7 depicts the spatially resolved local density of states (LDOS) of all coatings on the α-$CsPbI_3$ reconstructed surface (CsI-T, i_{PbI_2}, i_{2PbI_2} and i_{4CsI}) models. The horizontal orange lines depict the level alignments of the VBM and CBM of coatings and perovskites. The vertical red lines are representative of the band gap of the perovskites and coatings. Due to the enormous computational cost of the HSE functional, these interface calculations were performed without SOC. In the main text of the manuscript, we explain how we add the SOC for $CsPbI_3$ to the band offsets.

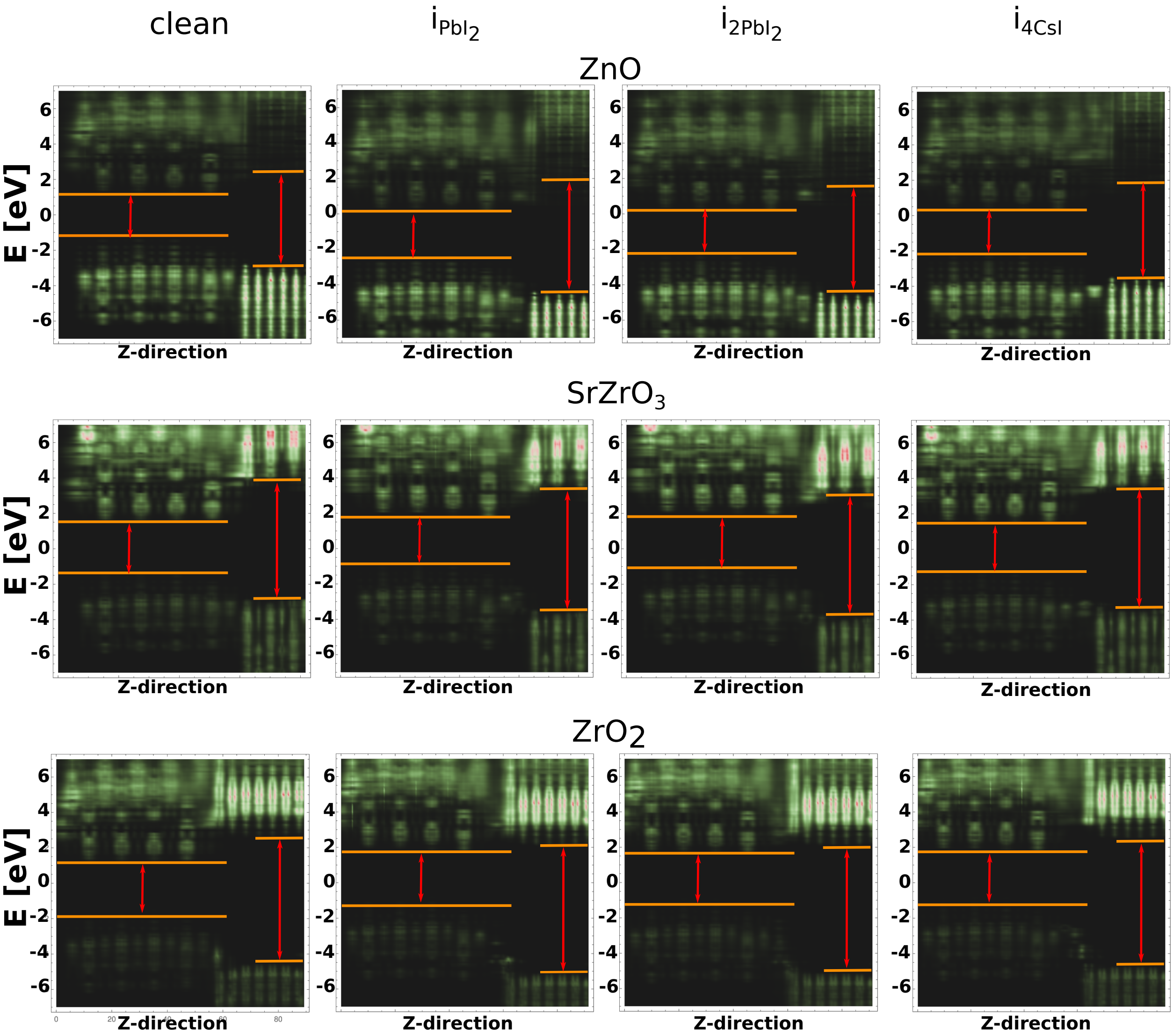

Figure S7: Spatially resolved local density of states of all coatings on the α-$CsPbI_3$ reconstructed surface (CsI-T, i_{PbI_2}, i_{2PbI_2} and i_{4CsI}) models. The horizontal orange lines depict VBM and CBM for the coatings and perovskites. The red vertical lines are representative of the energy band gap of the reconstructed α-$CsPbI_3$ surface models and coatings.
